# The Leading Impact Journal in Dermatopathology

**DOI:** 10.3390/dermatopathology11010012

**Published:** 2024-03-06

**Authors:** Gürkan Kaya

**Affiliations:** 1 Department of Dermatology, University Hospital of Geneva, 1205 Geneva, Switzerland; gkaya@hcuge.ch; 2 Department of Clinical Pathology, University Hospital of Geneva, 1205 Geneva, Switzerland

As the Editor-in-Chief of *Dermatopathology* and the President of the European Society of Dermatopathology (ESDP), I have the great pleasure of celebrating the 10th anniversary of *Dermatopathology*, the only online journal in the field of cutaneous pathology and the official journal of the ESDP. The year 2023 was a year full of hard-work and success for this well-established journal, which was rewarded by the highest ranking Impact Factor (IF: 1.9) among the journals in the field of dermatopathology, a well-deserved achievement!

It is very gratifying to see that *Dermatopathology*, since its first launch in 2014, has come all this way to obtain, after 9 years of intense work, its first IF and became a top journal in its field.

Since the beginning, *Dermatopathology* has aimed to provide a great quality of published articles with stringent controls, from the very first submission of abstracts with a content compatibility check to the selection of reviewers according to the subject/specialty match, the checking of the reviewer comments and the verification of the conformity of the modifications performed by the authors. These steps are controlled effectively by a meticulous work conducted by the Editor-in-Chief and the Editorial Office. The reviewers’ high-standard impact has increased the quality of the published articles. All these factors have played a major role in this remarkable success.

In 2024, we will continue with this hard work, maintain the high quality standards for *Dermatopathology* and try to do better with an IF higher than 2023! We will also continue to be active in online educational activity with *Dermatopathology* webinars, the first one of which was organized in 2023, and online courses, and be more visible in international scientific meeting platforms of dermatology, pathology and dermatopathology.

I would like to thank all the distinguished members of the Editorial Board, the Associate Editors, the reviewers, the Editorial Office and other Editorial team members in MDPI, the ESDP Board members, the ESDP members and the authors for their tremendous work and help in 2023, and for their contribution to make this journal one of the best journals of its kind.

